# Weight loss might be an early clinical manifestation of undiagnosed cancer: a nation-based cohort study

**DOI:** 10.1051/bmdcn/2018080425

**Published:** 2018-11-26

**Authors:** Shih-Wei Lai, Cheng-Li Lin, Kuan-Fu Liao

**Affiliations:** 1 College of Medicine, China Medical University Taichung 404 Taiwan; 2 Department of Family Medicine, China Medical University Hospital Taichung 404 Taiwan; 3 Management Office for Health Data, China Medical University Hospital Taichung 404 Taiwan; 4 College of Medicine, Tzu Chi University Hualien 970 Taiwan; 5 Division of Hepatogastroenterology, Department of Internal Medicine, Taichung Tzu Chi Hospital Taichung 427 Taiwan

**Keywords:** Weight loss, Cancer, Taiwan National Health Insurance Program

## Abstract

Background/Aim: No published nation-based study has examined the relationship between weight loss and cancer in Taiwan. The aim of this study was to investigate whether weight loss is an early clinical manifestation of cancer in Taiwan.

Methods: We conducted a nation-based, retrospective cohort study that analyzed the database of the Taiwan National Health Insurance Program. There were 8210 subjects aged 20 to 84 years with newly diagnosed weight loss selected as the weight loss group from 2000 to 2012, and 32826 randomly selected subjects without weight loss as the non-weight loss group. The weight loss and non-weight loss groups were matched along sex, age, and comorbidities. The incidence of cancer at the end of 2013 was examined in both groups.

Results: The weight loss group had a significantly higher incidence of cancer than the non-weight loss group during the first 3 months of follow-up (25.1 *vs.* 8.39 per 1000 person-years, with an incidence rate ratio 2.99, 95% CI 2.82, 3.18). The multivariable Cox proportional hazards regression model revealed that the adjusted HR of cancer was 1.05 for the weight loss group (95% CI 1.04, 1.05) as compared with the non-weight loss group.

Conclusion: Weight loss is associated with a small but statistically significant increase in the hazard of cancer. Weight loss might be an early clinical manifestation of undiagnosed cancer. Physicians should keep in mind the possibility of cancer when people present with weight loss and other possible cancer-related symptoms, particularly during the first 3 months of follow-up.

## 1. Introduction

Cancer remains a major public health concern globally due to its high prevalence and mortality. The Global Burden of Disease Cancer Collaboration estimated that there could have been as many as 17.5 million cancer cases in the world and 8.7 million deaths in 2015.[1] Cancer is one of the most common causes related to weight loss.[2, 3] The clinical definition of weight loss is a loss of 5% or more of one’s original body weight within 6 months.[3, 4] Previous case-series studies have revealed that, in addition to cancer-specific symptoms, weight loss may be the most frequent but non-specific complaint among patients with undiagnosed cancer.[5–7]

According to a report by the Ministry of Health and Welfare in Taiwan, cancer remained the first leading cause of death in 2016.[8] To the best of our knowledge, no published nation- based study examines the relationship between weight loss and cancer. From a perspective of preventive medicine, cancer can be intervened early only by early detection. If weight loss is proven to be an early clinical manifestation of undiagnosed cancer in Taiwan, physicians can keep in mind the possibility of cancer when people present with weight loss and other possible cancer-related symptoms. Thus, cancer can be detected early and early treatment can be performed. Therefore, we aimed to examine (1) whether weight loss is associated with cancer in Taiwan, and (2) how soon cancer can be detected after presentation of weight loss.

## 2. Methods

### 2.1 Study design and data source

Our study design and data source were adapted from previous studies.[9, 10] In brief, Taiwan is an independent country with 23 million people.[11–13] We conducted a nation-based, retrospective cohort study to analyze the database of the Taiwan National Health Insurance Program. This program was launched on March 1^st^, 1995, and it covers about 99.6% of the 23 million people living in Taiwan.[14]

### 2.2 Selection of subjects

Subjects aged 20 to 84 years with newly diagnosed weight loss from 2000 to 2012 were selected as the weight loss group (the International Classification of Diseases (ICD) 9th Revision, ICD-9 codes 783.21). For each subject with weight loss, approximately 4 subjects without weight loss were randomly selected as the non-weight loss group. The index date was defined as the date of weight-loss subjects being diagnosed with weight loss. Both weight loss and non-weight loss groups were matched according to sex, age (every 5-year interval), comorbidities, and the year of index date. Subjects with a history of any cancer before the index date were excluded from the study.

### 2.3 Potential comorbidities

Comorbidities which could be potentially related to cancer were adapted from previous studies and included alcohol-related disease, chronic obstructive pulmonary disease, diabetes mellitus, as well as chronic liver diseases including cirrhosis, hepatitis B infection, hepatitis C infection, and other chronic hepatitis. [15–19]

### 2.4 Major outcome

The major outcome was a new diagnosis of cancer (ICD-9 codes 140-208) during the follow-up period. All study subjects were followed until they were diagnosed with cancer or until the end of 2013.

### 2.5 Statistical analysis

We compared the differences of sex, age, and comorbidities between the weight loss and non-weight loss groups by a *Chi-square* test for categorized variables, and the f-test for continuous variables. The incidence of cancer was estimated as the event number of cancer identified during the follow-up period, divided by the total follow-up person-years for each group. At first, all variables were examined in an univariable model. In the next step, variables which were found to be statistically significant in the univariable model were further included in a multivariable model. A multivariable Cox proportional hazards regression model was used to examine the hazard ratio (HR) and 95% confidence interval (CI) for the association of cancer with weight loss. All analyses were performed using the SAS 9.2 (SAS Institute Inc., Carey, North Carolina, USA). The results were considered statistically significant when two-tailed *P* values were less than 0.05.

## 3. Results

### 3.1 Baseline information of the study population

Table 1 reveals the baseline information of the study population. There were 8210 subjects in the weight loss group and 32826 subjects in the non-weight loss group, with similar distributions of sex and age. The mean ages (standard deviation) of the study subjects were 54.3 (16.4) years for the weight loss group and 54.0 (16.5) years for the non-weight loss group (f-test, *P* = 0.11), without a statistic significance. There was no statistically significant difference of comorbidities between the weight loss and nonweight loss groups (*Chi*-square test, *P* > 0.05).

**Table 1:** Baseline information of subjects with and without weight loss.

Variable	Non-weight loss N = 32826		Weight loss N = 8210		
N	(%)	n	(%)	*P* value*
Sex					0.99
Female	15644	(47.7)	3913	(47.7)	
Male	17182	(52.3)	4297	(52.3)	
Age group (years)					0.98
20-39	7262	(22.1)	1808	(22.0)	
40-64	16095	(49.0)	4033	(49.1)	
65-84	9469	(28.9)	2369	(28.9)	
Age (years), mean (standard deviation)†	54.0	(16.5)	54.3	(16.4)	0.11
Baseline comorbidities					
Alcohol-related disease	1341	(4.09)	338	(4.12)	0.90
Chronic obstructive pulmonary disease	6356	(19.4)	1592	(19.4)	0.95
Chronic liver disease	8965	(27.3)	2242	(27.3)	0.99
Diabetes mellitus	2675	(8.15)	672	(8.19)	0.91

Data are presented as the number of subjects in each group with percentages given in parentheses, or mean with standard deviation given in parentheses.

* *Chi*-square test, and

† *t*-test comparing subjects with and without weight loss.

### 3.2 Incidence of cancer of the study population stratified by sex, age, and follow-up period

Table 2 reveals that the overall incidence of cancer was 2-fold higher in the weight loss group than that in the non-weight loss group (17.8 *vs.* 8.89 per 1000 person-years, 95% CI 1.89, 2.13). The incidences of cancer, as stratified by sex, age, and follow-up period, were all significantly higher in the weight loss group than in the non-weight loss group. Subjects aged 65 to 84 years in the weight loss group had a particularly higher incidence of cancer (36.5 per 1000 person-years). During the follow-up period, the weight loss group had a significantly higher incidence of cancer than the non-weight loss group in the first 3 months (25.1 *vs.* 8.39 per 1000 person-years, incidence rate ratio 2.99, 95 % CI 2.82, 3.18). Particularly, subjects in the weight loss group had the highest incidence of cancer during the first one month when compared with subjects in the non-weight loss group (50.2 *vs.* 8.59 per 1000 person-years, incidence rate ratio 5.84, 95% CI 5.48, 6.23).

**Table 2:** Incidence of cancer estimated by sex, age, and follow-up period between subjects with and without weight loss.

Variable	Non-weight loss				Weight loss				IRR*	(95% CI)
	N	Event	Person-years	Incidence‡	N	Event	Person-years	Incidence‡		
All	32826	1367	15756	8.89	8210	643	36080	17.8	2.00	(1.89, 2.13)
Sex										
Female	15644	493	74363	6.63	3913	234	17839	13.1	1.98	(1.81, 2.17)
Male	17182	874	79392	11.0	4297	409	18242	22.4	2.04	(1.88, 2.21)
Age group (years)										
20-39	7262	44	36352	1.21	1808	26	8929	2.91	2.41	(2.08, 2.79)
40-64	16095	590	75973	7.77	4033	286	18089	15.8	2.04	(1.87, 2.22)
65-84	9469	733	41430	17.7	2369	331	9062	36.5	2.06	(1.86, 2.29)
Follow-up period (months)										
< 1	32826	279	32493	8.59	8210	390	7776	50.2	5.84	(5.48, 6.23)
1-3	32133	450	65380	6.88	7611	126	16293	7.73	1.12	(1.05, 1.20)
³ 3	23188	638	66906	9.54	5870	127	15549	8.17	0.86	(0.78, 0.94)

‡ Incidence rate: per 1000 person-years.

* IRR (incidence rate ratio): weight loss *vs.* non-weight loss. (95% confidence interval)

### 3.3 Association of cancer with weight loss

Table 3 reveals the association of cancer with weight loss of the study population. After adjusting for covariables, the multivariable Cox proportional hazards regression model revealed that the adjusted HR of cancer was 1.05 (95 % CI 1.04, 1.05) for the weight loss group as compared to the non-weight loss group.

**Table 3:** Hazard ratio and 95% confidence interval of cancer associated with weight loss and comorbidities

Variable	Crude	Adjusted†	
	HR (95% CI)	HR	(95% CI)
Sex (male *vs.* female)	1.66(1.52, 1.82)	2.06	(1.87, 2.26)
Age (per one year)	1.05(1.04, 1.05)	1.51	(1.38, 1.66)
Weight loss (yes *vs.* no)	1.99(1.81, 2.19)	1.05	(1.04, 1.05)
Baseline comorbidities (yes *vs.* no)			
Alcohol-related disease	1.86(1.56, 2.23)	1.81	(1.50, 2.18)
Chronic obstructive pulmonary disease	1.96(1.79, 2.16)	0.98	(0.89, 1.08)
Chronic liver disease	1.55(1.42, 1.70)	1.34	(1.22, 1.47)
Diabetes mellitus	2.59(2.31, 2.90)	1.63	(1.45, 1.83)

† Variables found to be statistically significant in the univariable model were further examined in the multivariable model. Adjusted for sex, age, alcohol-related disease, chronic obstructive pulmonary disease, chronic liver disease, and diabetes mellitus.

## 4. Discussion

In this nation-based, retrospective cohort study, we found that the overall incidence of cancer was 2-fold greater in the weight loss group than in the non-weight loss group. The incidence of cancer in the weight loss group was higher during the first 3 months of follow-up, particularly in the first one month (50.2 *vs.* 8.59 per 1000 person-years, Table 2). That is, the greatest risk of cancer is within 1-3 months following coding of weight loss. In the Methods Section, we mentioned that subjects who had a history of any cancer before the index date were excluded from the study. Thus, weight loss indeed preceded a confirmed diagnosis of cancer. From the perspective of the high accessibility of the medical system for people in Taiwan, if the underlying etiology of weight loss is cancer, it does not need to take 3 months to make a confirmed diagnosis of cancer after the onset of weight loss and other cancer-related symptoms. Therefore, weight loss might be an early clinical manifestation of undiagnosed cancer in Taiwan. We suggest that physicians should keep in mind the possibility of cancer when people present with weight loss and other cancer-related symptoms particularly during the first 3 months of follow-up. Early detection of cancer means that early treatment can be performed. We also found that the cancer risk in the weight loss group distinctly decreased after 3 months when compared to the non-weight loss group (incidence rate ratio 0.86, Table 2). This means that weight loss may not be related to cancer but related to other etiologies. Therefore, if cancer is not detected in the first 3 months, watchful surveillance is highly recommended instead of blind diagnostic testing that may yield little useful information.[2, 4, 20]

## 5. Limitation

Several limitations of the study should be discussed. First, due to the inherent limitation of the database used, other possible cancer-related symptoms were not documented. We think that if people present with weight loss and other possible cancer-related symptoms, the likelihood of diagnosing cancer may be elevated. Similarly, if other possible cancer-related symptoms were present at the time of weight loss, we can further arrange a related investigation. Second, due to the same limitation, the status of alcohol consumption and cigarette smoking were not documented. We could only include alcohol-related disease and chronic obstructive pulmonary disease in our adjusting. Similarly, other risk factors related to specific cancers could not be included for adjustment. Third, weight loss is defined by using the ICD-9 code 783.21 “Loss of weight”. Due to this limitation, there is no discrimination between involuntary and voluntary weight loss. This limitation is not unique to this study and reflects a weakness in coding hierarchies. Furthermore, people who looked for medical help for weight loss are those anxious of the underlying etiologies. Therefore, we truly believe that their weight loss should be involuntary. Likewise, no further information is given about the amount of weight lost. This could be useful to move this field of research forward. The loss of 5% or more of original body weight within 6 months that is quoted as “the clinical definition of weight loss” in the background is based on reviews that say there is no consensus on this.[3, 4] Fourth, due to the same limitation, we could not discriminate the patients from primary care, secondary care, or a mixture of the two settings. Our analysis could not be stratified by or restricted to one setting. This makes a great difference to the interpretation of our findings. Moreover, it indicates a further research direction on this issue. Fifth, the outcome of cancer is defined by ICD-9 codes 140-208. This includes malignant, in- situ cancer, and unspecified cancer types, across all cancer sites. Theoretically, we should stratify the analysis to cancer site or cancer stage, but the sample size is a limitation and the effect is reduced when stratified. This indicates a further research direction on this issue. Sixth, many diseases can cause weight loss, such as hyperthyroidism, pulmonary tuberculosis, peptic ulcer disease, inflammatory bowel disease, depression, eating disorders, adrenal insufficiency, and others. It is difficult to include all confounders in adjusting.

## 6. Strength

Despite not being a novel topic, to the best of our knowledge, this is the first nation-based study to examine the relationship between weight loss and cancer in Taiwan. The study is methodologically sound and generally well-written. The results are impressive. It provides useful data to its readers.

## 7. Conclusion

Weight loss is associated with a small but statistically significant increase in the hazard of cancer. We think weight loss might be an early clinical manifestation of undiagnosed cancer. Physicians should give more attention to the possibility of undiagnosed cancer when people present with weight loss and other possible cancer-related symptoms, particularly during the first 3 months of follow-up.

## Specific author contribution statement

Shih-Wei Lai contributed to the conception of the article, initiated the draft of the article, and revised the article. Cheng-Li Lin conducted the data analysis and revised the article. Kuan-Fu Liao participated in the data interpretation and revised the article.

## Conflict of interest statement

The authors disclose no conflicts of interest.

## Ethical statement

The insurance reimbursement claims data used in this study were available for public access. Patient identification numbers were scrambled to ensure confidentiality. Patient informed consent was not required. This study was approved by the Research Ethics Committee of China Medical University and Hospital in Taiwan (CMUH-104-REC2-115).
